# Aqueous DMSO Mediated Conversion of (2-(Arylsulfonyl)vinyl)iodonium Salts to Aldehydes and Vinyl Chlorides

**DOI:** 10.3390/molecules21081073

**Published:** 2016-08-16

**Authors:** Eman Zawia, Wesley J. Moran

**Affiliations:** Department of Chemistry, University of Huddersfield, Queensgate, Huddersfield HD1 3DH, UK; eman.zawia@hud.ac.uk

**Keywords:** hypervalent iodine, iodonium salt, solvolysis, substitution

## Abstract

Vinyl(aryl)iodonium salts are useful compounds in organic synthesis but they are under-utilized and their chemistry is under-developed. Herein is described the solvolysis of some vinyl(phenyl)iodonium salts, bearing an arylsulfonyl group, in aqueous DMSO leading to aldehyde formation. This unusual process is selective and operates under ambient conditions. Furthermore, the addition of aqueous HCl and DMSO to these vinyl(aryl)iodonium salts allows their facile conversion to vinyl chlorides.

## 1. Introduction

Hypervalent iodine chemistry has received considerable attention in recent years, particularly in the area of small molecule synthesis [1,2,3]. Reasons for this include the ease-of-use of hypervalent iodine reagents, their low toxicity and the ability to effect useful, novel synthetic transformations.

Iodonium salts are iodine(III) species (also known as λ^3^-iodanes) with two carbon ligands and one non-carbon ligand [4]. These compounds are of increasing importance in synthesis, however, the majority of reported research in this area deals with the utility of diaryliodonium salts [5]. Alkenyl(aryl)iodonium salts are also useful reagents in synthesis, but their chemistry is relatively underdeveloped [6,7].

Alkenyl(aryl)iodonium salts are readily prepared from vinyl metal compounds, including silanes [8], stannanes [9], boronates/boronic acids [10], and zirconiums [11], by metal-iodine(III) exchange and from alkynyl(aryl)iodonium salts by conjugate addition of nucleophiles under protic conditions [12,13]. Additionally, hypervalent iodine reagents are known to undergo addition reactions to alkynes to generate alkenyl(aryl)iodonium salts [14,15]. These iodonium salts are reactive compounds due to the exquisite leaving group ability of the aryliodonium moiety coupled with its highly electron-withdrawing nature [16]. Reactions with nucleophiles can proceed through either S_N_V1 pathways [17], i.e., via vinylic cationic intermediates, or through S_N_2 mechanisms [18]. In the latter case, S_N_Vσ and S_N_Vπ mechanisms are possible, leading to inversion or retention of configuration respectively.

**Scheme 1:**
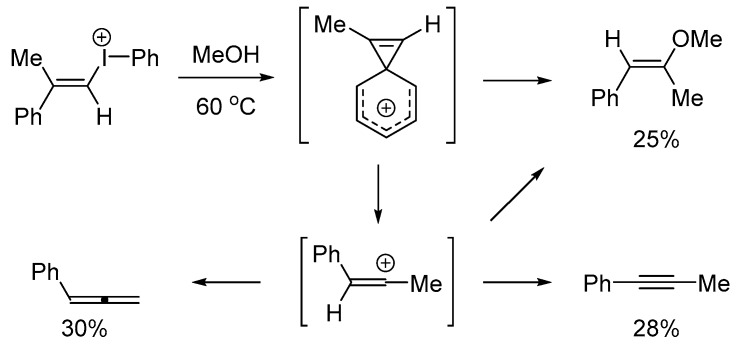
Solvolysis of (*Z*)-2-phenyl-1-propenyl(phenyl)iodonium tetrafluoroborate **1** with MeOH at 60 °C.

One particular reaction of alkenyl(aryl)iodonium salts is solvolysis which has been shown to occur in protic solvents such as methanol [19]. Typically, primary vinylic substrates require elevated temperatures for complete conversion and several products are formed. For example, heating (*Z*)-2-phenyl-1-propenyl(phenyl)iodonium tetrafluoroborate **1** in methanol at 60 °C leads to the formation of three products in approximately equal yields (Scheme 1). Deuterium labelling studies suggest that this process proceeds through phenyl-assisted loss of iodobenzene followed by addition of solvent, 1,2-migration or loss of a proton. Further work demonstrated that the outcome of this solvolysis process is dependent on the structure of the iodonium salt and the solvent used.

Hinkle and co-workers studied the fragmentation of similar β,β-disubstituted alkenyl(aryl)iodonium triflates in methanol solution and observed similar results to the tetrafluoroborate salts [20]. They also observed decomposition of the iodonium triflates upon standing in CDCl_3_ at room temperature over several hours. Okuyama and Ochiai reported the acetolysis of (*E*)-styryl- and (*E*)-1-decenyl(phenyl)iodonium tetrafluoroborate at elevated temperature [21].

Another process that alkenyl(aryl)iodonium salts have been shown to undergo is conversion to vinyl halides upon treatment with tetrabutylammonium halide in dichloromethane [22]. This transformation proceeds with retention of configuration.

During our studies on β-sulfone substituted alkenyl(phenyl)iodonium salts we noticed that upon standing in wet deuterated DMSO, the water was slowly consumed and the iodonium salt was converted into a new chemical entity. We decided to investigate this process.

## 2. Results and Discussion

**Scheme 2:**
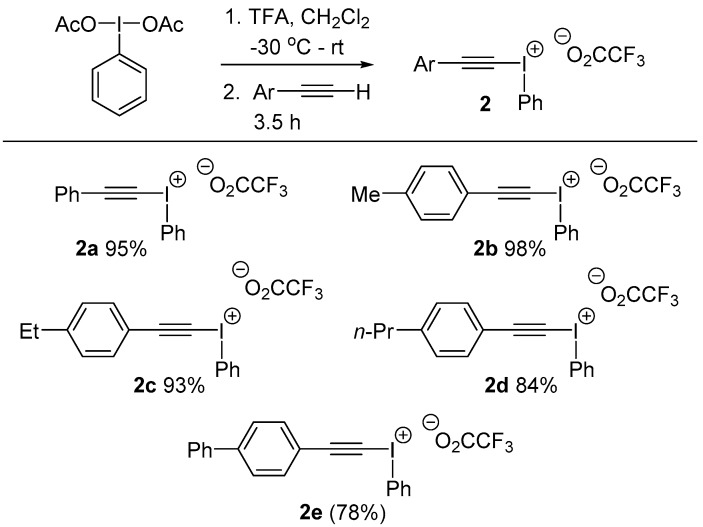
Preparation of alkynyl(phenyl)iodonium trifluoroacetates **2**.

We initiated our study with the preparation of a range of alkynyl(phenyl)iodonium trifluoroacetates **2**, which were prepared in one step from the terminal acetylenes following Carroll’s method (Scheme 2) [23]. High yields were obtained in all cases and recrystallization provided analytically pure samples, apart from **2e** which appeared to be somewhat unstable. **2e** was taken forward to the next step immediately before further decomposition could occur.

**Scheme 3:**
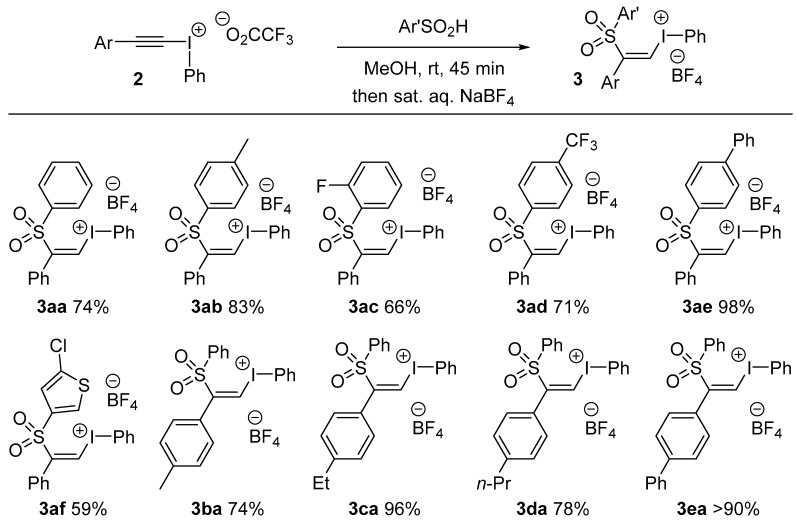
Preparation of alkenyl(phenyl)iodonium tetrafluoroborates **3**.

These alkynyl(phenyl)iodonium trifluoroacetates **2** were converted into a small family of alkenyl(phenyl)iodonium tetrafluoroborates **3** by mixing with aryl sulfinic acids in methanol (Scheme 3). As expected, only one isomer of product was formed in these reactions. These iodonium salts are completely stable in CDCl_3_ unlike those in Hinkle’s study [14]; possibly due to a stabilizing interaction between the sulfone oxygen and the iodine(III) center. With these compounds in hand, their reactivity in DMSO was investigated.

Iodonium salt **3aa** was dissolved in anhydrous DMSO and no observable change occurred after stirring overnight at room temperature (Table 1). However, when water was added to the solution the iodonium salt was slowly consumed and aldehyde **4aa** was formed along with unknown compounds in ratios that changed with each repetition (entry 2). We varied the solvent but no conversion of **3aa** occurred in all cases (entries 3–6). We avoided the use of alcohol solvents because of the previous studies highlighted above. Returning to the use of DMSO as solvent, we hypothesized that acidic or basic conditions could have an effect on this process. In the event, the addition of 2 M NaOH solution provided clean access to aldehyde **4aa** (entry 7). Subsequently, we found that simply shaking the reaction mixture with added water containing the aldehyde and unknown compounds led to complete conversion to the aldehyde **4aa** (entry 8). Presumably, the unknown compounds are intermediates en route to **4aa** and the increased agitation completes the solvolysis process. Performing the reaction with added hydrochloric acid led to complete conversion to the vinyl chloride **5aa** with retention of stereochemistry (entry 9).

**Scheme 4:**
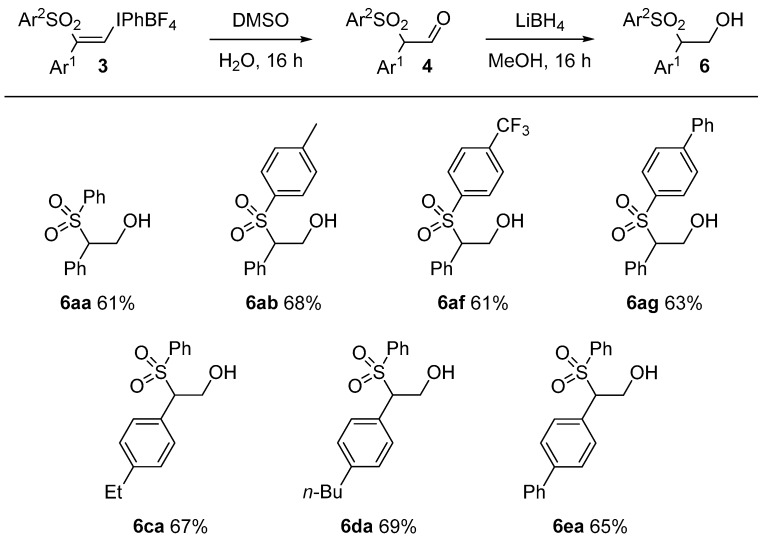
Conversion of iodonium salts **3** to aldehydes **4** and reduction to alcohols **6**.

The scope of this solvolysis reaction was investigated and aldehydes **4** were immediately reduced to the alcohols **6** to facilitate isolation of analytically pure compounds (Scheme 4). In all cases, yields of 60%–70% were obtained for the two steps. The tetrafluoroborate counterion is not critical to this process as the triflate analog of **3aa** underwent similarly facile hydrolysis. However, (*E*)-phenyl(styryl)- and (*E*)-1-octen-1-yl(phenyl)iodonium tetrafluoroborate, vinyliodonium salts lacking the β-sulfone moiety, were stable under these conditions and were returned unchanged.

**Scheme 5:**
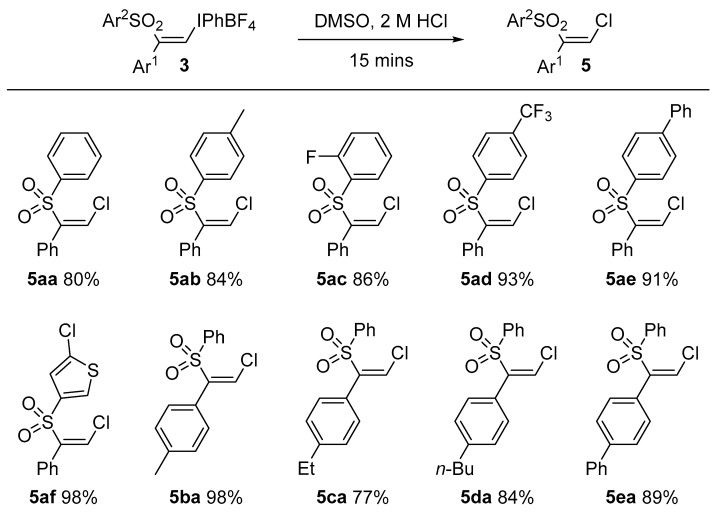
One-pot conversion of iodonium salts **3** to vinyl chlorides **5**.

The conversion of the iodonium salts **3** to vinyl chlorides **5** was investigated next and excellent yields were obtained in all cases (Scheme 5). Remarkably, this functional group conversion was complete within 15 min at room temperature. But again, (*E*)-phenyl(styryl)- and (*E*)-1-octen-1-yl(phenyl)iodonium tetrafluoroborate, vinyliodonium salts lacking the β-sulfone moiety, were stable under these conditions and were returned unchanged. As detailed above, this transformation has already been demonstrated using alternative conditions but we wished to determine whether DMSO was critical in our case (as with the aldehyde conversion). In the event, attempting the chlorination with 2 M HCl in a range of solvents, including dichloromethane, ethyl acetate, nitromethane, diethyl ether and acetonitrile, led to low conversions (5%–40%) in all cases after stirring overnight.

It is clear that DMSO has properties that aid the degradation of vinyliodonium salts and care should be taken that perfectly anhydrous solvent is used when this phenomenon is not desirable.

In summary, the solvolysis of alkenyl(phenyl)iodonium tetrafluoroborates containing a β-sulfone moiety occurs readily in aqueous DMSO solution at room temperature leading to selective aldehyde formation. Furthermore, the addition of aqueous HCl to the DMSO solution allows rapid vinyl chloride formation. This particular decomposition of vinyliodonium salts is unprecedented and demonstrates the unique properties of DMSO as a solvent.

## 3. Materials and Methods

### 3.1. General Information

All commercially available materials were used as received. ^1^H-NMR spectra were recorded at 400 MHz and ^13^C-NMR spectra at 100 MHz. IR spectra were recorded with neat samples and signals are labelled as strong (s), medium (m) or weak (w). HRMS were recorded in ESI mode and only molecular masses are reported. **2a** and **2b** were prepared according to the method of Carroll and co-workers and the data matched the literature values [23]. **3aa** was prepared following Ochiai’s protocol [14]. **6aa** and **6ab** are known compounds [24].

### 3.2. General Procedure for Alkynyl(phenyl)iodonium Trifluoroacetate ***2*** Formation: Preparation of ((4-Ethylphenyl)ethynyl)(phenyl)iodonium 2,2,2-trifluoroacetate ***2c***

Following Carroll’s procedure [23], trifluoroacetic acid (1.4 mL, 19 mmol, 2 equiv.) was added dropwise to a stirred solution of diacetoxyiodobenzene (3 g, 9.3 mmol, 1 equiv.) in CH_2_Cl_2_ (60 mL) at −30 °C. The mixture was stirred for 30 min, then warmed to room temperature and stirred for a further hour. 1-Ethyl-4-ethynyl benzene (1.3 mL, 9.3 mmol, 1 equiv.) was added dropwise and the resulting mixture was stirred in darkness at room temperature for 3.5 h. The solution was concentrated in vacuo to around 30 mL then diethyl ether (20 mL) and petroleum ether (40 mL) were added which initiated crystallization of the product. After cooling in a freezer (−20 °C) for 48 h, the solid was filtered and dried in vacuo to provide **2c** as a white crystalline solid (3.8 g, 93%). Melting point: 81–83 °C. IR: 1656 (s), 1417 (m), 1124 (s), 989 (m), 720 (s) cm^−1^. ^1^H-NMR: δ 1.22 (3H, t, *J* = 7.7 Hz), 2.67 (2H, q, *J* = 7.6 Hz), 7.20 (2H, d, *J* = 8.1 Hz), 7.39 (2H, d, *J* = 8.1 Hz), 7.49 (2H, t, *J* = 7.9 Hz), 7.60 (1H, t, *J* = 7.5 Hz) 8.15 (2H, d, *J* = 8.0 Hz). ^13^C-NMR: δ 15.5, 29.3, 44.8, 105.0, 115.8 (1C, q, *J* = 292 Hz), 114.4, 117.3, 117.7, 121.1, 128.6 (2C), 132.2, 132.4 (2C), 133.3 (2C), 133.7 (2C), 148.1, 162.9 (1C, q, *J* = 38 Hz). HRMS: *m*/*z* calcd. for [M − OCOCF_3_]^+^ C_18_H_18_I^+^ 333.0135 found 333.0140.

*((4-Butylphenyl)ethynyl)(phenyl)iodonium 2,2,2-trifluoroacetate,* **2d**: Melting point: 83–85 °C. IR: 1665 (s), 1417 (m), 1183 (s), 991.2 (m), 721 (s) cm^−1^. ^1^H-NMR: δ 0.91 (3H, t, *J* = 7.3 Hz), 1.33 (2H, hex, *J* = 7.3 Hz), 1.57 (2H, pen, *J* = 7.7 Hz), 2.63 (2H, t, *J* = 7.6 Hz), 7.18 (2H, d, *J* = 7.9 Hz), 7.39 (2H, d, *J* = 7.9 Hz), 7.50 (2H, t, *J* = 7.9Hz), 7.60 (1H, t, *J* = 7.4 Hz), 8.16 (2H, d, *J* = 8.4 Hz). ^13^C-NMR: δ 14.2, 22.6, 33.6, 36.0, 44.9, 105.0, 115.7 (1C, q, *J* = 291 Hz), 117.7, 121.0, 129.1 (2C), 132.1 (2C), 132.1 (2C), 132.4, 133.1 (2C), 133.6 (2C), 146.8, 162.9 (1C, q, *J* = 38 Hz). HRMS: *m*/*z* calcd. for [M − OCOCF_3_]^+^ C_16_H_14_I^+^ 361.448 found 361.01463.

*([1,1′-Biphenyl]-4-ylethynyl)(phenyl)iodonium 2,2,2-trifluoroacetate,* **2e**: This compound is unstable and complete characterization could not be obtained. ^1^H-NMR: δ (2H, d, *J* = 6.9 Hz), 7.33–7.61 (5H, m), 7.62–7.89 (5H, m), 8.17 (2H, d, *J* = 7.7 Hz). HRMS: *m*/*z* calcd. for [M − OCOCF_3_]^+^ C_20_H_14_I^+^ 381.0135 found 381.0132.

### 3.3. General Procedure for Alkenyl(phenyl)iodonium Tetrafluoroborate ***3*** Formation: Preparation of (Z)-Phenyl(2-phenyl-2-tosylvinyl)iodonium Tetrafluoroborate, ***3ab***

Following Ochiai’s procedure [14], phenyl(phenylethynyl)iodonium trifluoroacetate (0.50 g, 1.2 mmol, 1 equiv.) dissolved in MeOH (5 mL) was added to a solution of 4-methylbenzene sulfinic acid (0.20 g, 1.3 mmol, 1.1 equiv.) in MeOH (2 mL) at 0 °C under a N_2_ atmosphere. The reaction mixture was stirred for 45 min then poured into saturated aqueous sodium tetrafluoroborate solution (5 mL). A white precipitate was formed which was filtered off and washed with CH_2_Cl_2_ (2 × 8 mL). The combined organic layers were washed with water, dried over magnesium sulfate, filtered and concentrated in vacuo. The residue was a clear, colourless, viscous oil. A 1:1 mixture of diethyl ether and petroleum ether (5 + 5 mL) was added to the residue, then the mixture was swirled and the solvent removed by decantation. This was repeated until a white solid was formed. This crude product was recrystallized from the minimum amount of CH_2_Cl_2_ with slow addition of petroleum ether. (*Z*)-Phenyl(2-phenyl-2-tosylvinyl)iodonium tetrafluoroborate **3ab** was isolated as a white microcrystalline solid (0.54 g, 83%). Melting point: 125–127 °C. IR: 1535 (w), 1294 (m), 1135 (m), 1076 (s), 627 (s) cm^−1^. ^1^H-NMR: δ 2.40 (3H, s), 7.28 (2H, d, *J* = 7.9 Hz), 7.36 (2H, t, *J* = 7.6 Hz), 7.40–7.49 (3H, m), 7.63–7.73 (4H, m), 7.82 (1H, t, *J* = 7.6 Hz), 8.33 (1H, s), 8.40 (2H, d, *J* = 7.9 Hz). ^13^C-NMR: δ 22.5, 116.9, 117.1, 129.7 (2C), 129.9 (2C), 130.5 (2C), 131.7 (2C), 131.8 (2C), 133.1 (2C), 133.7, 134.1 (2C), 137.6, 146.9, 148.1. HRMS: *m*/*z* calcd. for [M − BF_4_]^+^ C_21_H_18_IO_2_S^+^ 461.0067 found 461.0079.

*(Z)-(2-((2-Fluorophenyl)sulfonyl)-2-phenylvinyl)(phenyl)iodonium tetrafluoroborate,* **3ac**: Melting point: 186–188 °C. IR: 1597 (w), 1474 (m), 1317 (m), 1022 (m), 522 (s) cm^−1^. ^1^H-NMR: δ 7.29–7.38 (4H, m), 7.40–7.46 (2H, t, m), 7.59 (1H, t, *J* = 9.6 Hz), 7.67 (2H, t, *J* = 7.8 Hz), 7.74–7.93 (3H, m), 8.40 (2H, d, *J* = 7.8 Hz), 8.55 (1H, s). ^13^C-NMR: δ 116.9, 118.4, 119.1 (1C, d, *J* = 20 Hz), 124.4 (1C, d, *J* = 13 Hz), 127.2, 130.1 (2C), 130.4 (2C), 131.3, 132.1 (1C, d, *J* = 5.4 Hz), 133.2 (2C), 134.2, 137.7 (2C), 140.5 (1C, d, *J* = 9.8 Hz), 146.6, 159.1, 161.6. HRMS: *m*/*z* calcd. for [M − BF_4_]^+^ C_20_H_15_FIO_2_S^+^ 464.9816 found 464.9826.

*(Z)-Phenyl(2-phenyl-2-((4-(trifluoromethyl)phenyl)sulfonyl)vinyl)iodonium tetrafluoroborate,* **3ad**: Melting point: 184–186 °C. IR: 1317 (s), 1129 (s), 1117 (m), 1059 (s), 641 (s) cm^−1^. ^1^H-NMR: δ 7.29 (2H, d, *J* = 7.8 Hz), 7.38 (2H, t, *J* = 7.6 Hz), 7.46 (1H, t, *J* = 7.6 Hz), 7.68 (2H, t, *J* = 7.8 Hz), 7.82 (1H, t, *J* = 7.4 Hz), 8.1 (4H, S), 8.39 (2H, d, *J* = 8.6 Hz), 8.50 (1H, s). ^13^C-NMR: 117.1, 119.2, 124.3 (1C, q, *J* = 274.2 Hz), 128.3 (1C, d, *J* = 3.3 Hz), 130.1 (2C), 130.7 (2C), 130.8 (2C), 131.3. 131.9, 133.0 (2C), 134.3 (2C), 136.1 (1C, q, *J* = 33 Hz), 137.6 (2C), 140.6, 145.8. HRMS: *m*/*z* calcd. for [M − BF_4_]^+^ C_21_H_15_F_3_IO_2_S^+^ 514.9784 found 514.9807.

*(Z)-(2-([1,1′-Biphenyl]-4-ylsulfonyl)-2-phenylvinyl)(phenyl)iodonium tetrafluoroborate,* **3ae**: Melting point: 162–164 °C. IR: 1596 (w), 1484 (m), 1289 (m), 1050 (m), 674 (s) cm^−1^. ^1^H-NMR: δ 7.33 (2H, d, *J* = 7.2 Hz), 7.38 (2H, t, *J* = 7.5 Hz), 7.42–7.58 (4H, m), 7.67 (2H, t, *J* = 7.9 Hz), 7.77 (2H, d, *J* = 7.8 Hz), 7.82 (1H, t, *J* = 7.5 Hz), 7.88 (2H, d, *J* = 8.2 Hz), 7.96 (2H, d, *J* = 8.5 Hz), 8.39 (1H, s), 8.41(2H, d, *J* = 7.8 Hz). ^13^C-NMR: δ 116.9, 117.7, 128.6 (2C), 129.1 (2C), 130.0 (2C), 130.4 (2C), 130.5 (2C), 130.6 (2C), 131.8 (2C), 131.9, 133.1, 134.1(2C), 135.3, 137.6(2C), 138.7, 146.7, 148.1. HRMS: *m*/*z* calcd. for [M − BF_4_]^+^ C_26_H_20_IO_2_S^+^ 523.0223 found 523.0236.

*(Z)-(2-((5-Chlorothiophen-2-yl)sulfonyl)-2-phenylvinyl)(phenyl)iodonium tetrafluoroborate,* **3af**: Melting point: 187–189 °C. IR: 1399 (w), 1310 (w), 639 (s), 628 (m), 523 (m) cm^−1^. ^1^H-NMR: δ 7.35 (2H, d, *J* = 7.6 Hz), 7.39–7.46 (3H, m), 7.51 (1H, t, *J* = 7.4 Hz), 7.65 (2H, t, *J* = 7.8 Hz), 7.73–7.83 (2H, m), 8.36 (3H, d, *J* = 9.4 Hz). ^13^C-NMR: 116.9, 117.2, 130.2 (2C), 130.9 (2C), 131.6, 131.9, 132.2, 133.2 (2C), 134.2, 134.6, 137.6 (2C), 139.0, 142.4, 147.3. HRMS: *m*/*z* calcd. for [M − BF_4_]^+^ C_18_H_13_ClIO_2_S_2_^+^ 486.9085 found 486.9091.

*(Z)-Phenyl(2-(phenylsulfonyl)-2-(p-tolyl)vinyl)iodonium,* **3ba**: Melting point: 125–127 °C. IR: 1535 (m), 1303 (m), 1136 (m), 1059 (s), 651 (s) cm^−1^. ^1^H-NMR: δ 2.26 (3H, s), 7.17 (4H, t, *J* = 6.9 Hz), 7.61–7.72 (4H, m), 7.77–7.89 (4H, m), 8.29 (1H, S), 8.39 (2H, d, *J* = 7.8 Hz). ^13^C-NMR: δ 22.07, 116.9, 128.9, 129.6 (2C), 130.4 (2C), 130.5 (2C), 131.2 (2C), 133.1(2C), 134.1, 136.8 (3C), 136.9, 137.6, 141.8, 146.7. HRMS: *m*/*z* calcd for [M − BF_4_]^+^ C_21_H_18_IO_2_S^+^ 461.0067 found 461.0082.

*(Z)-(2-(4-Ethylphenyl)-2-(phenylsulfonyl)vinyl)(phenyl)iodonium,* **3ca**: Melting point: 122–124 °C. IR: 1526 (m), 1299 (m), 1134 (m), 1060 (s), 620 (s) cm^−1^. ^1^H-NMR: δ 1.10 (3H, t, *J* = 7.5 Hz), 2.56 (2H, q, *J* = 7.8 Hz), 7.20 (4H, s), 7.67 (4H, t, *J* = 7.6 Hz), 7.85–7.97 (4H, m), 8.31 (1H, S), 8.39 (2H, d, *J* = 3.8 Hz). ^13^C-NMR: δ 16.4, 29.1, 116.9, 117.0, 129.1, 129.3 (2C), 129.5 (2C), 130.5 (2C), 131.2 (2C), 133.1 (2C), 134.1, 136.7, 136.9, 137.6 (2C), 146.7, 147.9. HRMS: *m*/*z* calcd. for [M − BF_4_]^+^ C_22_H_20_IO_2_S^+^ 475.0223 found 475.0247.

*(Z)-(2-(4-Butylphenyl)-2-(phenylsulfonyl)vinyl)(phenyl)iodonium tetrafluoroborate,* **3da**: Melting point: 120–122 °C. IR: 1526 (m), 1293 (m), 1130 (s), 1048 (s), 569 (s) cm^−1^. ^1^H-NMR: δ 0.84 (3H, t, *J* = 7.5 Hz), 1.21 (2H, hex, *J* = 7.5 Hz), 1.77 (2H, pen, *J* = 7.5 Hz), 2.49–2.55 (2H, m), 7.18 (4H, s), 7.66 (4H, q, *J* = 7.2 Hz), 7.81 (4H, t, *J* = 8.6 Hz) 8.31 (1H, S), 8.39 (2H, d, *J* = 3.9 Hz). ^13^C-NMR: δ 14.9, 22.7, 33.9, 35.6, 116.8, 116.9, 129.01, 129.5, 129.8 (2C), 130.4 (2C), 131.1 (2C), 133.03 (2C), 134.1, 136.7 (2C), 136.9, 137.6 (2C), 146.5, 146.7. HRMS: *m*/*z* calcd. for [M − BF_4_]^+^ C_24_H_24_IO_2_S^+^ 503.0536 found 503.0549.

*(Z)-(2-([1,1′-Biphenyl]-4-yl)-2-(phenylsulfonyl)vinyl)(phenyl)iodonium,* **3ea**: Melting point: 164–166 °C. IR: 1501 (w), 1310 (m), 1132 (m), 1073 (m), 533 (s) cm^−1^. ^1^H-NMR: δ 7.40 (3H, t, *J* = 8.5 Hz), 7.47 (2H, t, *J* = 7.6 Hz), 7.62–7.73 (8H, m), 7.83 (2H, t, *J* = 7.2 Hz), 7.80 (2H, d, *J* = 7.8 Hz), 8.41 (3H, t, *J* = 7.3 Hz). ^13^C-NMR: δ 117.0, 117.8, 127.9 (2C), 128.0 (2C), 129.5, 129.6 (2C), 130.3 (2C), 130.7, 131.13 (2C), 131.3 (2C), 131.9, 133.1 (2C), 134.1, 136.8, 137.1, 137.6, 139.7, 143.2, 146.3. HRMS: *m*/*z* calcd. for [M − BF_4_]^+^ C_26_H_20_IO_2_S^+^ 523.0223 found 523.0207.

### 3.4. General Procedure for Formation of Alcohols ***6***: Preparation of 2-phenyl-2-(phenylsulfonyl)ethan-1-ol, ***6aa***

(*Z*)-Phenyl(2-phenyl-2-(phenylsulfonyl)vinyl)iodonium tetrafluoroborate, **3aa** (50 mg, 0.094 mmol, 1 equiv.) was dissolved in DMSO (0.5 mL) at room temperature. Deionized water (20 μL) was added and the mixture was stirred overnight. Water (2.5 mL) was added to the mixture and the solution was shaken and then extracted with ethyl acetate (2 × 5 mL). The organic layer was dried with anhydrous magnesium sulfate, filtered and concentrated under vacuum. The crude product was dissolved in methanol (2 mL) and LiBH_4_ (2 mg, 0.094 mmol, 1 equiv.) was added in one portion. The mixture was stirred overnight and then extracted with diethyl ether (2 × 5 mL) and washed with water (5 mL). The residue was purified by flash chromatography (5:1 petrolem ether/EtOAc) which furnished **6aa** as a colorless oil (0.015 g, 61%). IR: 3521 (w), 2428 (s), 1235 (m), 567 (w) cm^−1^. ^1^H-NMR: δ 2.77 (OH, dd, *J* = 8.7, 4.8 Hz), 4.16 (1H, ddd, *J* = 13, 8.7, 4.5 Hz), 4.38 (1H, dd, *J* = 7.9, 4.7 Hz), 4.66 (1H, ddd, *J* = 12, 7.9, 4.5 Hz), 7.06 (2H, d, *J* = 7.4 Hz), 7.22–7.30 (3H, m), 7.34 (1H, t, *J* = 7.4 Hz), 7.59–7.70 (4H, m). ^13^C-NMR: δ 61.5, 73.3, 126.1, 126.2, 129.2 (2C), 129.8 (2C), 129.9 (2C), 130.0 (2C), 130.6, 140.7. HRMS: *m*/*z* calcd. for [M + H]^+^ C_14_H_1__5_O_3_S^+^ 263.0736 found 263.0701.

*2-Phenyl-2-tosylethan-1-ol,* **6ab**: IR: 3521 (w), 2428 (s), 1235 (m), 567 (w) cm^−1^. ^1^H-NMR: δ 2.40 (3H, s), 2.99 (OH, br), 4.08 (1H, ddd, *J* = 13, 7.8, 4.9 Hz), 4.33 (1H, dd, *J* = 8.2, 4.5 Hz), 4.61 (1H, t, *J* = 10 Hz), 7.03 (2H, d, *J* = 7.9 Hz ), 7.19 (2H, d, *J* = 7.4 Hz), 7.21–7.27 (2H, m), 7.38 (2H, d, *J* = 8.2 Hz). ^13^C-NMR: δ 21.9, 61.7, 73.0, 128.9 (2C), 129.3 (2C), 129.4 (2C), 129.7 (2C), 129.9 (2C), 131.3, 134.0, 145.4. HRMS: *m*/*z* calcd. for [M + H]^+^ C_15_H_17_O_3_S^+^ 277.0893 found 277.0876.

*2-Phenyl-2-((4-(trifluoromethyl)phenyl)sulfonyl)ethan-1-ol,* **6af**: IR: 2359 (w), 1319 (s), 1172 (s), 712 (m) cm^−1^. ^1^H-NMR: δ 2.79 (OH, br), 4.16 (1H, d, *J* = 12 Hz), 4.38 (1H, dd, *J* = 7.9, 4.7 Hz), 4.66 (1H, dd, *J* = 12, 7.9 Hz), 7.06 (2H, d, *J* = 7.5 Hz ), 7.27 (2H, t, *J* = 7.4 Hz), 7.34 (1H, t, *J* = 7.4 Hz), 7.61–7.70 (4H, m). ^13^C-NMR: δ61.4, 73.3, 123.4 (1C, q, *J* = 271.4 Hz), 126.2 (1C, d, *J* = 3.4 Hz), 129.2 (2C), 129.8 (2C), 129.9 (2C), 130.0 (2C), 130.6, 135.7, 136.0, 140.8. HRMS: *m*/*z* calcd. for [M + NH_4_]^+^ C_15_H_17_F_3_NO_3_S^+^ 348.0876 found 348.0877.

*2-([1,1′-Biphenyl]-4-ylsulfonyl)-2-phenylethan-1-ol,* **6ag**: IR: 3565 (w), 2359 (s), 2250 (m), 650 (w) cm^−1^. ^1^H-NMR: δ 2.92–3.00 (OH, m), 4.07–4.19 (1H, m), 4.39 (1H, dd, *J* = 8.3, 4.5 Hz), 4.61–4.71 (1H, m), 7.08 (1H, d, *J* = 7.5 Hz), 7.26 (4H, t, *J* = 7.2 Hz), 7.33 (1H, t, *J* = 7.2 Hz), 7.38–7.52 (3H, m), 7.53–7.65 (5H, m). ^13^C-NMR: δ 61.7, 73.2, 127.6 (2C), 127.7 (2C), 129.0 (2C), 129.1, 129.4 (2C), 129.6, 129.9 (2C), 130.0 (2C), 131.2, 135.5, 139.2, 147.2. HRMS: *m*/*z* calcd. for [M + H]^+^ C_20_H_22_O_3_S^+^ 356.1315 found 356.1322.

*2-(4-Ethylphenyl)-2-(phenylsulfonyl)ethan-1-ol,* **6ca**: IR: 2561 (s), 1345 (m), 1227 (m), 1162 (s), 767 (m) cm^−1^. ^1^H-NMR: δ 1.19 (3H, t, *J* = 7.7), 2.61 (2H, q, *J* = 7.7 Hz), 2.91 (OH, dd, *J* = 9.0 HZ, 4.7 Hz), 4.09 (1H, ddd, *J* = 13, 9, 5 Hz), 4.33 (1H, dd, *J* = 8.2, 4.3 Hz), 4.59 (1H, ddd, *J* = 12, 8.0, 4.0 Hz), 6.94 (2H, d, *J* = 8.1 Hz ), 7.07 (2H, d, *J* = 7.8 Hz), 7.41 (2H, t, *J* = 7.8 Hz), 7.53 (2H, d, *J* = 7.6 Hz), 7.59 (1H, t, *J* = 7.4 Hz). ^13^C-NMR: δ 15.8, 28.9, 61.7, 72.9, 128.2, 128.5 (2C), 129.1 (2C), 129.4 (2C), 129.8 (2C), 134.3 (2C), 137.1, 145.9. HRMS: *m*/*z* calcd. for [M + NH_4_]^+^ C_16_H_22_NO_3_S^+^ 308.1315 found 308.1318.

*2-(4-Butylphenyl)-2-(phenylsulfonyl)ethan-1-ol,* **6da**: IR: 2341 (s), 1446 (m), 1304 (m), 1142 (s), 687.6 (m) cm^−1^. ^1^H-NMR: δ 0.91 (3H, t, *J* = 7.3 Hz), 1.24–1.35 (2H, m), 1.54 (2H, pent, *J* = 7.4 Hz), 2.56 (2H, t, *J* = 7.6 Hz), 2.99 (OH, br), 4.09 (1H, dd, *J* = 8.1, 4.4 Hz), 4.33 (1H, dd, *J* = 7.9, 4.6 Hz), 4.59 (1H, dd, *J* = 12, 8.1 Hz), 6.92 (2H, d, *J* = 7.9 Hz), 7.04 (2H, d, *J* = 7.4 Hz), 7.39 (2H, t, *J* = 7.8 Hz), 7.51 (2H, d, *J* = 3.8 Hz), 7.60 (1H, t, *J* = 7.4 Hz). ^13^C-NMR: δ 14.2, 22.5, 33.7, 35.6, 61.6, 72.8 128.1, 129.0 (3C), 128.9, 129.4, 130.4, 129.7, 134.2, 137.1, 144.5. HRMS: *m*/*z* calcd. for [M + NH_4_]^+^ C_18_H_26_NO_3_S^+^ 336.1628 found 336.1630.

*2-([1, 1′-Biphenyl]-4-yl)-2-(phenylsulfonyl)ethan-1-ol,* **6ea**: IR: 3568 (w), 2358 (s), 2248 (m), 647 (w) cm^−1^. ^1^H-NMR: δ 4.1–4.2 (1H, m), 4.40 (1H, dd, *J* = 8.1, 4.5 Hz), 4.65 (1H, dd, *J* = 12, 8.1 Hz), 7.11 (2H, d, *J* = 7.7 Hz), 7.37 (1H, t, *J* = 7.3 Hz), 7.40–7.51 (6H, m), 7.52–7.64 (5H, m). ^13^C-NMR: δ 61.6, 72.8, 127.4 (2C), 127.6 (2C), 128.2, 129.2 (2C), 129.3 (2C), 129.4 (2C), 130.1 (2C), 130.4, 134.4, 137.2, 140.4, 142.4. HRMS: *m*/*z* calcd. for [M + Na]^+^ C_20_H_18_NaO_3_S^+^ 361.0869 found 361.0869.

### 3.5. General Procedure for Formation of Vinyl Chlorides ***5***: Preparation of (Z)-(2-Chloro-1-(phenylsulfonyl)vinyl)benzene, ***5aa***

(*Z*)-Phenyl(2-phenyl-2-(phenylsulfonyl)vinyl)iodonium tetrafluoroborate **3aa** (0.050 g, 0.094 mmol) was dissolved in DMSO (0.5 mL) at room temperature. HCl (2 N, 50 μL) was added and the mixture was stirred for 15 min. Brine (5 mL) was added to the mixture and the solution was shaken. This was extracted with ethyl acetate (2 × 10 mL) and washed with water (5 mL). The organic layer was dried with anhydrous magnesium sulfate, filtered and concentrated under vacuum. The residue was purified by flash chromatography (20:1 petrolem ether/EtOAc) which furnished **5aa** as a colorless oil (0.026 g, 80%). IR: 1586 (w), 1444 (m), 1320 (m), 1147 (s), 628 (s) cm^−1^. ^1^H-NMR: δ 6.73 (1H, s), 7.26 (2H, d, *J* = 7.3 Hz), 7.34 (2H, t, *J* = 7.2 Hz), 7.41 (1H, t, *J* = 7.2 Hz), 7.49 (2H, t, *J* =7.8 Hz), 7.62 (1H, t, *J* = 7.4 Hz), 7.83 (2H, d, *J* = 7.6 Hz). ^13^C-NMR: δ 128.7 (2C), 128.8 (2C), 129.2, 129.4 (2C), 130.0, 130.5, 133.2 (2C), 134.2, 140.5, 145.6. HRMS: *m*/*z* calcd. for [M + H]^+^ C_14_H_11_ClO_2_S^+^ 279.0232 found 279.0241.

*(Z)-1-((2-Chloro-1-phenylvinyl)sulfonyl)-4-methylbenzene,* **5ab**: IR: 1578 (m), 1443 (m), 1146 (s), 1083 (m), 754 (s) cm^−1^. ^1^H-NMR: δ 2.42 (3H, s), 6.69 (1H, s), 7.26 (4H, d, *J* = 16 Hz), 7.33 (2H, t, *J* = 7.4 Hz), 7.35 (1H, t, *J* = 7.4 Hz), 7.70 (2H, d, *J* = 7.7 Hz). ^13^C-NMR: δ 22.7, 128.7 (2C), 128.8, 128.9 (2C), 129.9 (3C), 130.5, 133.3 (2C), 137.5, 145.2, 145.7. HRMS: *m*/*z* calcd. for [M + H]^+^ C_15_H_14_ClO_2_S^+^ 293.0398 found 293.0390.

*(Z)-1-((2-Chloro-1-phenylvinyl)sulfonyl)-2-fluorobenzene,* **5ac**: IR: 1599 (w), 1264 (m), 1146 (m), 1076 (s), 770 (m) cm^−1^. ^1^H-NMR: δ 6.82 (1H, s), 7.16 (1H, t, *J* = 9.0 Hz), 7.29 (1H, t, *J* = 7.6 Hz), 7.32–7.43 (5H, m), 7.56–7.65 (1H, m), 7.99 (1H, dd, *J* = 7.5, 1.6 Hz). ^13^C-NMR: δ 117.2 (1C, d, *J* = 20 Hz), 124.7 (1C, d, *J* = 3.8 Hz), 128.8 (3C), 129.4 (1C, d, *J* = 1.2 Hz), 130.1 (2C), 130.4 (1C, d, *J* = 1.2 Hz), 131.3, 132.5, 136 (1C, d, *J* = 8.5 Hz), 145.9, 160.0 (1C, d, *J* = 256 Hz). HRMS: *m*/*z* calcd. for [M + NH_4_]^+^ C_14_H_14_ClFNO_2_S^+^ 314.0412 found 314.0410.

*(Z)-1-((2-Chloro-1-phenylvinyl)sulfonyl)-4-(trifluoromethyl)benzene,* **5ad**: IR: 1591 (m), 1320 (s), 1125 (s), 1085 (s), 714 (m) cm^−1^. ^1^H-NMR: δ 6.80 (1H, s), 7.27 (2H, d, *J* = 8.5 Hz), 7.33–7.47 (3H, m), 7.76 (2H, d, *J* = 7.9 Hz), 7.96 (2H, d, *J* = 7.97 Hz). ^13^C-NMR: δ 123.5 (1C, q, *J* = 274 Hz), 126.4, 128.9 (2C), 129.1 (2C), 130.3 (2C), 130.5 (2C), 132.6, 135.8 (1C, q, *J* = 33 Hz), 143.9, 144.9. HRMS: *m*/*z* calcd. for [M + NH_4_]^+^ C_15_H_14_ClF_3_NO_2_S^+^ 364.0380 found 364.0375.

*(Z)-4-((2-Chloro-1-phenylvinyl)sulfonyl)-1,1′-biphenyl,* **5ae**: IR: 1592 (w), 1329 (m), 1148 (m), 1085 (w), 581 (m) cm^−1^. ^1^H-NMR: δ 6.75 (1H, s), 7.30 (2H, d, *J* = 3.7 Hz), 7.36 (2H, t, *J* = 7.6 Hz), 7.42 (2H, t, *J* = 7.8 Hz), 7.48 (2H, t, *J* = 7.6 Hz), 7.60 (2H, d, *J* = 7.6 Hz), 7.70 (2H, d, *J* = 8.3 Hz), 7.88 (2H, d, *J* = 8.3 Hz). ^13^C-NMR: δ 127.7 (2C), 127.9 (2C), 128.8 (2C), 129.1, 129.2 (2C), 129.4, 129.5 (2C), 130.0, 130.6 (2C), 133.2, 138.9, 139.4, 145.6, 147.1. HRMS: *m*/*z* calcd. for [M + H]^+^ C_20_H_19_ClNO_2_S^+^ 372.0820 found 372.0816.

*(Z)-2-Chloro-5-((2-chloro-1-phenylvinyl)sulfonyl)thiophene,* **5af**: IR: 1402 (m), 1332 (s), 1146 (s), 990 (m), 605 (s) cm^−1^. ^1^H-NMR: δ 6.78 (1H, s), 6.93 (1H, d, *J* = 3.9 Hz), 7.29 (2H, d, *J* = 7.9 Hz), 7.38 (2H, t, *J* = 7.7 Hz) 7.40–7.46 (2H, m). ^13^C-NMR: δ 127.4, 128.8 (2C), 129.8, 130.2, 130.5 (2C), 132.6, 134.6, 138.9, 140.5, 145.2. HRMS: *m*/*z* calcd. for [M + NH_4_]^+^ C_12_H_12_Cl_2_NO_2_S_2_ 335.9681 found 335.9684.

*(Z)-1-(2-Chloro-1-(phenylsulfonyl)vinyl)-4-methylbenzene,* **5ba**: IR: 1578 (m), 1443 (m), 1146 (s), 1083 (m), 754 (s) cm^−1^. ^1^H-NMR: δ 2.36 (3H, s), 6.70 (1H, s), 7.15 (4H, s), 7.49 (2H, t, *J* = 7.5 Hz), 7.61(1H, t, *J* = 7.5 Hz), 7.83 (2H, d, *J* = 7.5 Hz). ^13^C-NMR: δ 21.7, 128.6 (2C), 128.9, 129.3 (2C), 129.5 (2C), 130.3, 130.4 (2C), 134.1, 140.2, 140.6, 145.5. HRMS: *m*/*z* calcd. for [M + NH_4_]^+^ C_15_H_17_ClNO_2_S^+^ 310.0663 found 310.0663.

*(Z)-1-(2-Chloro-1-(phenylsulfonyl)vinyl)-4-ethylbenzene,* **5ca**: IR: 2359 (s), 1709 (m), 1332 (m), 1149 (s), 1084 (m), 722 (m) cm^−1^. ^1^H-NMR: δ 1.23 (3H, t, *J* = 7.7 Hz), 2.65 (2H, q, *J* = 7.6 Hz), 6.70 (1H, s), 7.18 (4H, s), 7.49 (2H, t, *J* = 7.7 Hz), 7.61(1H, t, *J* = 7.6 Hz), 7.83 (2H, d, *J* = 7.7 Hz). ^13^C-NMR: δ 15.6, 28.9, 128.3 (2C), 128.6 (2C), 128.9, 129.2 (2C), 130.4, 130.5 (2C), 134.1, 140.6, 145.6, 146.4. HRMS: *m*/*z* calcd. for [M + NH_4_]^+^ C_16_H_19_ClNO_2_S^+^ 324.0820 found 324.0822.

*(Z)-1-(2-Chloro-1-(phenylsulfonyl)vinyl)-4-propylbenzene,* **5da**: IR: 2359 (s), 1709 (m), 1332 (m), 1149 (s), 1084 (m), 722 (m) cm^−1^. ^1^H-NMR: δ 0.93 (3H, t, *J* = 7.3 Hz), 1.34 (2H, hex, *J* = 7.3 Hz), 1.59 (2H, q, *J* = 7.6 Hz), 2.61 (2H, t, *J* = 7.9 Hz), 6.70 (1H, s), 7.10–7.20 (4H, m), 7.48 (2H, t, *J* = 7.6 Hz), 7.60 (1H, t, *J* = 7.4 Hz), 7.83 (2H, d, *J* = 8.1 Hz). ^13^C-NMR: δ 15.6, 28.9, 128.3 (2C), 128.6 (2C), 128.9, 129.2 (2C), 130.4, 130.5 (2C), 134.1, 140.6, 145.6, 146.4. HRMS: *m*/*z* calcd. for [M + H]^+^ C_18_H_20_ClO_2_S 335.0867 found 335.0866.

*(Z)-4-(2-Chloro-1-(phenylsulfonyl)vinyl)-1,1′-biphenyl,* **5ea**: IR: 1481 (w), 1318 (m), 1180 (m), 1004 (w), 648 (s) cm^−1^. ^1^H-NMR: δ 6.78 (1H, s), 7.32–7.41 (3H, m), 7.42–7.54 (4H, m), 7.55–7.67 (5H, m), 7.88 (2H, d, *J* = 7.6 Hz). ^13^C-NMR: δ 127.4 (2C), 127.5 (2C), 128.2, 128.6 (2C), 129.3 (2C), 129.3 (2C), 129.4, 130.9 (2C), 132.0, 134.2, 140.3, 140.5, 142.9, 145.3. HRMS: *m*/*z* calcd. for [M + NH_4_]^+^ C_20_H_19_ClNO_2_S^+^ 372.0820 found 372.0821.

## Figures and Tables

**Table 1:**
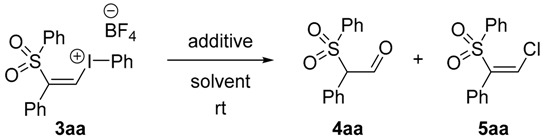
Effect of solvent and additives on reactivity of iodonium salt **3aa**.

Entry	Additive	Solvent	Conversion/% ^a^
1	None	DMSO	0
2	2 equiv. H_2_O	DMSO	100 (25%–75% **4aa**)
3	2 equiv. H_2_O	MeCN	0
4	2 equiv. H_2_O	MeNO_2_	0
5	2 equiv. H_2_O	EtOAc	0
6	2 equiv. H_2_O	Et_2_O	0
7	2 equiv. 2 M NaOH	DMSO	100 (**4aa**)
8	2 equiv. H_2_O ^b^	DMSO	100 (**4aa**)
9	2 equiv. 2 M HCl	DMSO	100 (**5aa**)

^a^ Determined by ^1^H-NMR analysis; ^b^ With vigorous shaking.

## Abbreviations

The following abbreviations are used in this manuscript:

DMSO	dimethyl sulfoxide
NMR	nuclear magnetic resonance
TFA	trifluoroacetic acid
M	molar
rt	room temperature
